# Urinary biomarkers for the early prediction of bronchopulmonary dysplasia in preterm infants: A pilot study

**DOI:** 10.3389/fped.2022.959513

**Published:** 2022-08-11

**Authors:** Xuewei Cui, Jianhua Fu

**Affiliations:** Department of Pediatrics, Shengjing Hospital of China Medical University, Shenyang, China

**Keywords:** 8-hydroxy-2’-deoxyguanosine, biomarkers, bronchopulmonary dysplasia, N-terminal pro-brain natriuretic peptide, preterm infants

## Abstract

**Background:**

This study investigated whether 8-hydroxy-2’-deoxyguanosine (8-OHdG) and N-terminal pro-brain natriuretic peptide (NT-proBNP) concentrations in the urine could predict bronchopulmonary dysplasia (BPD) in preterm infants.

**Methods:**

This prospective cohort study enrolled 165 preterm infants, of whom 70 developed BPD. We measured urinary 8-OHdG and NT-proBNP concentrations from day of life (DOL) 7 to 28. Then, we evaluated the prediction efficiency by receiver operating characteristic curves and assessed correlations between the two biomarkers. Finally, we identified the predictive risk factors for BPD by multivariable logistic regression.

**Results:**

8-OHdG and NT-proBNP levels were significantly higher from DOL 7 to 28 in the BPD group than in the control group (*P* < 0.05). Additionally, the 8-OHdG level was positively correlated with the NT-proBNP level (r: 0.655–0.789, *P* < 0.001), and the 8-OHdG and NT-proBNP levels were positively correlated with mechanical ventilation duration and oxygen exposure time (r: 0.175–0.505, *P* < 0.05) from DOL 7 to 28. Furthermore, the 8-OHdG (DOL 14–28) and NT-proBNP (DOL 7–28) levels were significantly associated with BPD development (*P* < 0.05).

**Conclusion:**

The urine 8-OHdG concentrations from DOL 14 to 28 and NT-proBNP concentrations from DOL 7 to 28 may be practical non-invasive predictors of BPD development in preterm infants.

## Introduction

Bronchopulmonary dysplasia (BPD) is a consequence of intrauterine lung development disruptions and postnatal lung injuries. Therefore, the younger the gestational age (GA), the higher the incidence of BPD (1). Despite improved perinatal care with prenatal corticosteroids, postnatal surfactants, and mild mechanical ventilation strategies, the BPD prevalence in neonatal intensive care units remains high (2, 3). Therefore, there is an urgent need to explore non-invasive, reliable, and convenient predictive biomarkers for early clinical detection and identification of BPD.

Currently, early BPD prediction methods include biomarker detection and predictive models based on machine learning. The known biomarkers include serum interleukin-6, carboxyhemoglobin, lipid hydroperoxide, and glutathione in serum, tracheal aspiration (TA) fluid, and bronchoalveolar lavage fluid (BALF) (4–6). Furthermore, the current early BPD predictive models are machine learning models that include perinatal factors, clinical information, genomics, proteomics, and metabolomics (7). However, repeated blood collection can damage the integrity of the skin and mucous membranes and increase the chance of infection in preterm infants, and obtaining BALF through a bronchoscope considerably increases a newborn’s pain. Furthermore, the predictive models require high detection technology, which is not conducive to daily clinical practice in primary hospitals. However, urine testing is convenient, non-invasive, inexpensive, and allows for repetitive measurements compared to blood and BLAF samples. Thus, it might be a better option.

Several studies have shown that oxidative stress is key in BPD occurrence (8). DNA damage is the most severe manifestation of oxidative stress, and 8-hydroxy-2’-deoxyguanosine (8-OHdG) is the most common DNA damage marker (9). Preterm infants have reduced antioxidant defenses in response to oxidative challenges because they generate reactive oxygen species (ROS) faster and have limited antioxidant protection (10). This oxidative-antioxidant imbalance mediates lung epithelial damage, leading to BPD (11). Therefore, preterm infants are more vulnerable to oxidative stress damage in the lungs, especially under long-term mechanical ventilation or oxygen support (12).

N-terminal pro-brain natriuretic peptide (NT-proBNP) is a highly stable, biologically inactive N-terminal fragment of B-type natriuretic peptide (BNP) secreted by cardiomyocytes with a low clearance rate. Furthermore, NT-proBNP is eliminated from the body through the liver and kidneys (13). Plasma NT-proBNP has been widely used to assess heart failure in adults, children, and neonates. Recent preterm infant studies found an association between elevated serum NT-proBNP concentrations and preterm birth complications, including respiratory distress syndrome (RDS), diaphragmatic hernia, and hemodynamically significant patent ductus arteriosus (PDA) (14–16). These complications are closely related to BPD development. Moreover, the NT-proBNP concentrations in blood parallel those in urine (17).

This prospective cohort study analyzed urine 8-OHdG and NT-proBNP levels in preterm infants and assessed their ability to predict BPD, thus acting as non-invasive biomarkers.

## Materials and methods

### Patient selection and bronchopulmonary dysplasia

We conducted a prospective, single-center cohort study on preterm infants admitted to the First Neonatal Ward of Shengjing Hospital of China Medical University from January 2021 to January 2022. Singleton, live birth infants with a GA of <32 weeks and birth weight of <1,500 g admitted to the neonatal ward in the first week after birth were included. Furthermore, all parents gave informed consent. The exclusion criteria were incomplete medical records, discharge or death before 36-week postmenstrual age, twins or multiple births, severe congenital heart disease, chromosomal disease, genetic metabolic disease, and other severe malformations.

BPD and severity were defined using the 2018 Workshop Diagnostic Consensus Criteria (18). The preterm infants were classified into control or BPD groups based on the presence or absence of BPD. The ethics committee of Shengjing Hospital of China Medical University reviewed and approved this study (ethical code: 2022PS389K).

### Sample collection and measurements

We obtained 1.5 mL of spot urine samples from the preterm infants in the morning on days of life (DOL) 7, 14, 21, and 28; the samples were stored at –80°C until use. After thawing at 20–26°C, the samples were centrifuged at 300 × *g* for 10 min at 4°C. Urinary 8-OHdG and NT-proBNP levels were measured using enzyme-linked immunosorbent assay kits (8-OHdG: cat#: CEA660Ge, Uscn Life Science Inc., Wuhan, P.R. China; NT-proBNP: cat#: SEA485Hu, Uscn Life Science Inc., Wuhan, P.R. China). We also measured urine creatinine (Cr) by spectrophotometry and a colorimetric creatinine assay kit (cat#: ab204537, Abcam, Cambridge, United Kingdom) to correct diuresis variability from fluid restriction and the application of caffeine and diuretics. The results were expressed as ng of 8-OHdG per mg of Cr [i.e., the urinary 8-OHdG/creatinine ratio (UDGCR), ng/mg Cr] and ng of NT-proBNP per mg of Cr [i.e., the urinary NT-proBNP/creatinine ratio (UNBCR), ng/mg Cr]. All samples were analyzed in duplicate.

### Statistical analyses

Statistical analyses were performed using SPSS (version 24.0; SPSS Inc., Chicago, IL, United States). Normally distributed continuous variables are presented as the mean ± standard deviations and compared using *t*-tests. Data with skewed distributions were expressed as medians (interquartile ranges), and comparisons between groups were performed using non-parametric tests. Categorical variables were described as numbers (percentages) and compared between groups using chi-square tests. We assessed the strength of the association between continuous variables using Spearman rank-order coefficients, and the Kolmogorov–Smirnov test was used to test whether the variables had a normal (Gaussian) distribution. The predictive values of urinary 8-OHdG and NT-proBNP levels were estimated using the areas under the receiver operating characteristic (ROC) curves. Non-normally distributed variables were logarithmically transformed before multiple logistic regression analysis, which was used to predict factors associated with BPD. Based on clinical significance, univariate analysis, and previous research, four multivariable regression models were conducted, and several confounders were chosen and modified to assess correlations between urine 8-OHdG and NT-proBNP levels and BPD. Statistical significance was set at *P* < 0.05. We registered this study on the website: http://www.chictr.org.cn/listbycreater.aspx (number ChiCTR2200057749).

## Results

### Baseline characteristics

We enrolled 238 preterm infants and included 165 in the analysis (control group = 95; BPD group = 70). The perinatal characteristics of the preterm infants did not differ between the two groups (*P* > 0.05; Table 1). The GA was younger, the birth weight was lower, and the total hospital stay was longer in the BPD group than in the control group (*P* < 0.001; Table 1). Furthermore, the mechanical ventilation duration, oxygen exposure time, and the retinopathy of prematurity (ROP), PDA, and pulmonary hypertension (PH) incidences significantly differed between the two groups (*P* < 0.01; Table 1).

**TABLE 1:** Perinatal and preterm infant characteristics.

Variable	Control group (*n* = 95)	BPD group (*n* = 70)	t/X^2^/Z	*P*-value
**Perinatal characteristics**				
Maternal age	30.09 ± 4.27	30.13 ± 3.71	0.053	0.958
Cesarean (*n*, %)	67 (70.5)	48 (68.6)	0.073	0.787
PROM > 18 h (*n*, %)	20 (21.1)	17 (24.3)	0.242	0.623
Antenatal steroid usage (*n*, %)	54 (56.8)	41 (58.6)	0.049	0.824
PIH (*n*, %)	35 (36.8)	29 (41.4)	0.357	0.550
GDM (*n*, %)	23 (24.2)	23 (32.9)	1.499	0.221
Chorioamnionitis (*n*, %)	23 (24.2)	26 (37.1)	3.229	0.072
**Preterm infant characteristics**				
GA (weeks)	29.63 ± 1.35	28.63 ± 1.54	4.354	<0.001***
Birth weight (g)	1290.22 ± 161.69	1114.04 ± 179.03	6.608	<0.001***
Male sex (*n*, %)	43 (45.3)	38 (54.3)	1.313	0.252
Apgar score (1 min)	7.00 (6.00,9.00)	7.00 (6.00,9.00)	−1.937	0.053
Apgar score (5 min)	10.00 (9.00,10.00)	10.00 (9.00,10.00)	-0.498	0.618
Surfactant therapy (n, %)	59 (62.1)	47 (67.1)	0.445	0.505
Total hospital stay (day)	47.00 (40.00,52.00)	56.00(48.00,69.25)	−5.425	<0.001***
Mechanical ventilation (day)	13.00 (7.00,22.00)	33.50(21.00,47.00)	−7.653	<0.001***
Oxygen consumption duration (day)	21.00 (10.00,28.00)	50.00(37.75,60.00)	−9.026	<0.001***
PDA (*n*, %)	28 (29.5)	35 (50.0)	7.194	0.007**
NEC (*n*, %)	10 (10.5)	13 (18.6)	2.174	0.140
EOS (*n*, %)	18 (18.9)	10 (14.3)	0.622	0.430
IVH (*n*, %)	19 (20.0)	23 (32.9)	3.511	0.061
ROP (*n*, %)	12 (12.6)	28 (40.1)	19.520	<0.001***
PH (*n*, %)	7 (7.4)	25 (35.7)	20.715	<0.001***

**P < 0.01; ***P < 0.001. BPD, bronchopulmonary dysplasia; EOS, early-onset sepsis; GA, gestational age; GDM, gestational diabetes mellitus; IVH, intraventricular hemorrhage; NEC, necrotizing enterocolitis; PDA, patent ductus arteriosus; PH, pulmonary hypertension; PIH, pregnancy-induced hypertension; ROP, retinopathy of prematurity; PROM, premature rupture of membranes.

### Urine 8-hydroxy-2’-deoxyguanosine and N-terminal pro-brain natriuretic peptide levels

**FIGURE 1:**
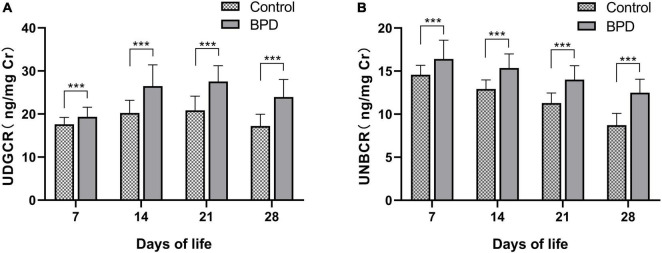
Urinary 8-OHdG **(A)** and NT-proBNP **(B)** levels between two groups. 8-OHdG, 8-hydroxy-2’-deoxyguanosine; NT-proBNP, N-terminal pro-brain natriuretic peptide; UNBCR, urinary NT-proBNP/creatinine ratio; UDGCR, urinary 8-OHdG/creatinine ratio. ***P < 0.001.

The 8-OHdG level was significantly higher in the BPD group than in the control group from DOL 7 (19.34 ± 2.24 vs. 17.63 ± 1.59 ng/mg Cr, *P* < 0.001; Figure 1A and Supplementary Table 1) to DOL 28 (23.95 ± 4.06 vs. 17.21 ± 2.75 ng/mg Cr, *P* < 0.001; Figure 1A and Supplementary Table 1). Likewise, the NT-proBNP concentrations were significantly higher in the BPD group than in the control group from DOL 7 (16.40 ± 2.19 vs. 14.57 ± 1.10 ng/mg Cr, *P* < 0.001; Figure 1B and Supplementary Table 1) to DOL 28 (12.50 ± 1.55 vs. 8.72 ± 1.37 ng/mg Cr, *P* < 0.001; Figure 1B and Supplementary Table 1). The urine NT-proBNP levels decreased from DOL 7 to DOL 28 in both groups (Figure 1B).

### Receiver operating characteristic curves

We used ROC curves to determine the usefulness of urine 8-OHdG and NT-proBNP levels as predictive markers for BPD. Both biomarkers had good predictive efficiency [i.e., area under the curve (AUC) > 0.7] on DOL 7, 14, 21, and 28. On DOL 28, 19.20 ng/mg Cr was the ideal cutoff value for 8-OHdG, which had the maximum diagnostic efficacy (AUC = 0.920, Youden index = 0.692, sensitivity 87.1%, specificity 82.1%, *P* < 0.001; Table 2), indicating a good predictive ability for BPD. However, the AUC for NT-proBNP on DOL 28 was 0.953 (cutoff value = 9.67 ng/mg Cr, Youden index = 0.800, sensitivity 100.0%, specificity 80.0%, *P* < 0.001: Table 2) which seemed to represent stronger predictive ability.

**TABLE 2:** UDGCR and UNBCR cutoff values for predicting BPD.

Variable	AUC	Cutoff (ng/mg Cr)	Sensitivity	Specificity	Youden index	*P*-value
UDGCR DOL 7	0.731	18.05	0.743	0.674	0.417	<0.001***
UDGCR DOL 14	0.876	22.79	0.900	0.821	0.721	<0.001***
UDGCR DOL 21	0.904	21.86	1.000	0.653	0.653	<0.001***
UDGCR DOL 28	0.920	19.20	0.871	0.821	0.692	<0.001***
UNBCR DOL 7	0.740	16.18	0.543	0.926	0.469	<0.001***
UNBCR DOL 14	0.889	14.99	0.686	0.989	0.675	<0.001***
UNBCR DOL 21	0.908	12.18	0.857	0.800	0.657	<0.001***
UNBCR DOL 28	0.953	9.67	1.000	0.800	0.800	<0.001***

***P < 0.001. AUC, area under the curve; BPD, bronchopulmonary dysplasia; DOL, day of life; UNBCR, urinary NT-pro-BNP/creatinine ratio; UDGCR, urinary 8-OHdG/creatinine ratio.

### Correlation analysis

**FIGURE 2:**
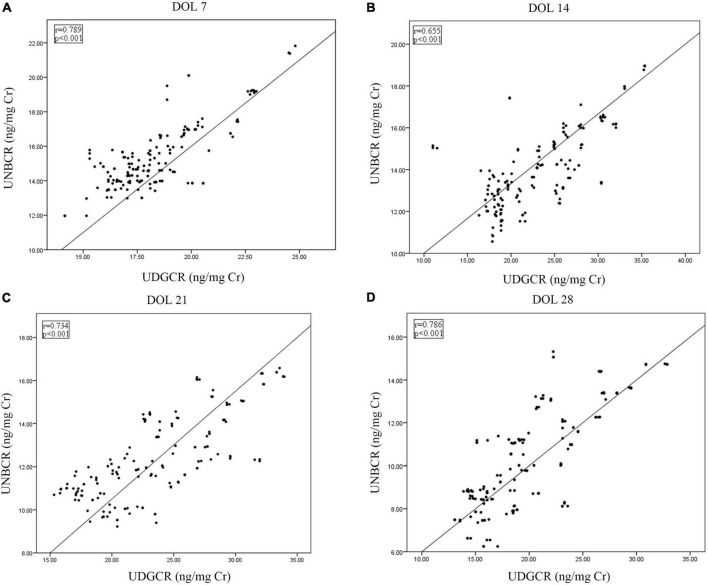
Correlations between urine 8-OHdG and NT-proBNP from DOL 7 to 28 **(A–D)**. 8-OHdG, 8-hydroxy-2’-deoxyguanosine; DOL, days of life; NT-proBNP, N-terminal pro-brain natriuretic peptide; UDGCR, urinary 8-OHdG/creatinine ratio; UNBCR, urinary NT-proBNP/creatinine ratio.

The urine 8-OHdG level positively correlated with the NT-proBNP level on DOL 7, 14, 21, and 28 (*P* < 0.001; Figures 2A–D). From DOL 7 to 28, the levels of 8-OHdG and NT-proBNP were additionally shown to be strongly associated with mechanical ventilation duration and oxygen exposure time (Spearman rank-order coefficient: 0.175–0.505, *P* < 0.05; Supplementary Table 2).

### Multivariable logistic regression analysis

After adjusting for potential confounding variables, the connection between UDGCR from DOL 14 to 28, UNBCR from DOL 7 to 28, oxygen exposure duration, and BPD maintained in the four multivariable logistic regression models. This finding indicated that urine 8-OHdG and NT-proBNP levels were independent risk factors for the development of BPD, with the strength of this association peaking at DOL 28 (odds ratio = 2.141 and 7.199, respectively, *P* < 0.01; Table 3).

**TABLE 3:** Predictors and risk factors for BPD (multivariable logistic regressions).

Variable	B	OR	95% CI	*P*-value
**DOL 7**				
UDGCR DOL 7	0.254	1.289	[0.803, 2.071]	0.293
UNBCR DOL 7	0.728	2.070	[1.141, 3.757]	0.017*
GA	0.424	1.529	[0.908, 2.573]	0.110
Birth weight	−0.002	0.998	[0.994, 1.002]	0.349
Mechanical ventilation duration	−0.013	0.987	[0.919, 1.060]	0.716
Oxygen exposure time	0.149	1.160	[1.087, 1.238]	0.000***
PDA	0.843	2.323	[0.734, 7.355]	0.152
**DOL 14**				
UDGCR DOL 14	0.277	1.319	[1.085, 1.605]	0.006**
UNBCR DOL 14	1.459	4.301	[1.923, 9.622]	0.000***
GA	0.186	1.205	[0.670, 2.166]	0.533
Birth weight	-0.002	0.998	[0.993, 1.004]	0.571
Mechanical ventilation duration	−0.039	0.962	[0.865, 1.070]	0.476
Oxygen exposure time	0.159	1.172	[1.062, 1.293]	0.002**
PDA	1.200	3.319	[0.648, 17.014]	0.150
**DOL 21**				
UDGCR DOL 21	0.416	1.515	[1.135, 2.023]	0.005**
UNBCR DOL 21	1.057	2.879	[1.485, 5.583]	0.002**
GA	0.269	1.308	[0.668, 2.562]	0.433
Birth weight	−0.003	0.997	[0.991, 1.004]	0.403
Mechanical ventilation duration	−0.008	0.992	[0.897, 1.098]	0.882
Oxygen exposure time	0.134	1.143	[1.050, 1.246]	0.002**
PDA	0.617	1.854	[0.396, 8.686]	0.433
**DOL 28**				
UDGCR DOL 28	0.761	2.141	[1.241, 3.692]	0.006**
UNBCR DOL 28	1.974	7.199	[1.868, 27.742]	0.004**
GA	0.452	1.571	[0.653, 3.778]	0.313
Birth weight	−0.002	0.998	[0.990, 1.006]	0.681
Mechanical ventilation duration	0.046	0.532	[0.907, 1.208]	0.532
Oxygen exposure time	0.200	1.221	[1.044, 1.428]	0.012*
PDA	1.529	4.613	[0.445, 47.811]	0.200

*P < 0.05; **P < 0.01; ***P < 0.001. BPD, bronchopulmonary dysplasia; CI, confidence interval; DOL, day of life; GA, gestational age; OR, odds ratio; PDA, patent ductus arteriosus; UNBCR, urinary NT-proBNP/creatinine ratio; UDGCR, urinary 8-OHdG/creatinine ratio.

## Discussion

This study is the first to prospectively measure urine 8-OHdG and NT-proBNP levels over time in very low and extremely low birth weight infants and evaluate associations with BPD development in the first 28 DOLs. This is also the first study to explore the correlation between urine 8-OHdG and NT-proBNP levels. We found significantly elevated 8-OHdG and NT-proBNP levels in the urine of preterm infants with BPD from DOL 7 to DOL 28 compared to those without BPD. There was also a positive correlation between the 8-OHdG and NT-proBNP levels from DOL 7 to 28. Furthermore, after adjusting for confounding factors, the 8-OHdG (DOL 14 to 28) and NT-proBNP (DOL 7–28) levels were significantly associated with BPD. We also utilized ROC curves to determine the optimum predictive cutoff values of urine 8-OHDG and NT-proBNP for BPD from DOL 7 to DOL 28. These results may help identify preterm infants with a high risk of BPD early and provide effective prevention and treatment measures.

Oxygen free radical-mediated diseases include RDS, PDA, BPD, ROP, necrotizing enterocolitis, intraventricular hemorrhage, and periventricular leukomalacia (19, 20). In addition, oxidative stress is an essential contributor to lung injury, beginning with acute inflammatory injury (as in RDS) and progressing to pulmonary microvascular remodeling and impaired alveolarization, leading to BPD (21, 22). 8-OHdG is a biomarker of endogenous oxidative stress-related DNA damage (23). Hsiao et al. (24) investigated the relationship between the 8-OHdG level in TA and BPD, reporting that the TA 8-OHdG level on postnatal day 28 was associated with BPD (*P*< 0.05). In addition, 8-OHdG is considerably water soluble and is excreted in the urine without further metabolism (25). Joung et al. (26) found that preterm infants with moderate to severe BPD had higher urinary levels of 8-OHdG than infants with no to mild BPD. As a result, we hypothesized that urine 8-OHdG might be a biological marker for non-invasive prediction of BPD.

Our results support this conjecture. It showed that the urine 8-OHdG level was significantly higher from DOL 7 to 28 in the BPD group than in the control group. Besides, our study also found that the urine 8-OHdG level positively correlated with the mechanical ventilation duration and oxygen exposure time on DOL 7–28. Most importantly, our multiple regression analysis demonstrated that urine 8-OHdG levels from DOL 14 to 28 were independently associated with a higher risk of BPD (odds ratio = 1.319, 1.515, and 2.141, respectively, *P* < 0.01) and could predict the development of BPD as early as DOL 14.

An increasing number of studies have supported serum NT-proBNP levels as a biomarker to forecast, diagnose, and manage respiratory diseases, including BPD (27–29). Furthermore, recent studies have identified associations between urine NT-proBNP concentrations and preterm birth-related complications, such as ROP, PDA, and PH (30–32).

Our prospective study demonstrated that the urine NT-proBNP concentrations were significantly elevated from DOL 7 to 28 in preterm infants who, many weeks later, developed BPD (*P* < 0.05). This result linked circulatory stress to BPD in the first 4 weeks of life for preterm infants and were consistent with serum NT-proBNP level trends reported by Song et al. (33). We also found that urine NT-proBNP levels from DOL 7 to 28 positively correlated with mechanical ventilation duration and oxygen exposure time. BPD is characterized by arrested alveolar development and pulmonary microvascular dysplasia. Prenatal exposure to oxidative stress, postnatal hyperoxia, and prolonged mechanical ventilation may increase pulmonary vascularization damage by modulating vascular tone (34). Elevated serum NT-proBNP levels may be related to raised pulmonary vascular pressure and diastolic dysfunction (33, 35). Our study discovered a persistently positive correlation between an oxidative stress-related biomarker (i.e., 8-OHdG) and NT-proBNP levels in urine from DOL 7 to 28 (r: 0.655–0.789, *P* < 0.001). Therefore, we hypothesized that increased NT-proBNP levels in preterm infants with BPD may be related to oxidative stress. A prospective observational study found that the plasma NT-proBNP level at DOL 28 had a moderate predictive value for BPD severity (36). Nevertheless, our study accelerated the forecast time to DOL 7. The results of the multiple logistic regression analyses showed that urine NT-proBNP levels between DOL 7 and 28 were substantially related to the development of BPD (odds ratio = 2.070–7.199, *P* < 0.05), and that BPD may be predicted in preterm infants as early as the first week after birth.

Our study had several limitations. First, this pilot study was conducted at a single center with 165 preterm infants. Second, owing to limited domestic detection methods, urine NT-proBNP levels were measured using a commercially available ELISA kit rather than the chemiluminescent sandwich immunoassay used in other studies (30, 37). Thus, variations in the measurement results from different detection techniques should be considered. Third, the PDA and PH incidences in our study were higher in the BPD group than in the control group, and it is unclear if the occurrence of PH and PDA affected the NT-proBNP levels in preterm infants with BPD in our study. Therefore, a multicenter, more extensive validation study is needed to clarify the value of urinary 8-OHdG and NT-proBNP for predicting and monitoring BPD in preterm infants.

## Conclusion

Our study confirmed a persistently positive relationship between urinary 8-OHdG and NT-proBNP levels from DOL 7 to 28. In addition, for the first time, we defined urine 8-OHdG and NT-proBNP cutoff values for predicting BPD from DOL 7 to 28. We also demonstrated that the 8-OHdG levels from DOL 14 to 28 and NT-proBNP levels from DOL 7 to 28 in the urine might be valuable non-invasive biomarkers for early predicting of BPD in preterm infants. Our findings provide foundational evidence for future multicenter studies with larger sample sizes. Finding reliable and convenient biomarkers to identify high-risk infants would improve early BPD detection and treatment.

## Data availability statement

The raw data supporting the conclusions of this article will be made available by the authors, without undue reservation.

## Ethics statement

The studies involving human participants were reviewed and approved by The Ethics Committee of Shengjing Hospital of China Medical University. Written informed consent to participate in this study was provided by the participants’ legal guardian/next of kin.

## Author contributions

XC and JF designed the study. XC drafted the manuscript, conducted the research, and collected and analyzed the data. JF critically reviewed the manuscript for intellectual content. Both authors assisted with manuscript preparation and approved the final version, and contributed to the study conception and design.
