# Supplementary material for: Urinary biomarkers for the early prediction of bronchopulmonary dysplasia in preterm infants: A pilot study
Source: Front Pediatr. 2022 Aug 11;10:959513. doi: 10.3389/fped.2022.959513 (PMC9403535; doi:10.3389/fped.2022.959513)
Supplement: Supplementary file 1 [file Table_1.doc]

**Title: Urinary Biomarkers for the Early Prediction of Bronchopulmonary Dysplasia in Preterm Infants: A Pilot Study**

**Supplementary Table 1**. Urinary 8-OHdG and NT-proBNP levels

| Variable | Control group (n=95) | BPD group  (n=70) | *P*-value |
| --- | --- | --- | --- |
| UDGCR DOL 7 | 17.63±1.59 | 19.34±2.24 | <0.001^***^ |
| UDGCR DOL 14 | 20.24±2.93 | 26.48±4.92 | <0.001^***^ |
| UDGCR DOL 21 | 20.86±3.28 | 27.55±3.66 | <0.001^***^ |
| UDGCR DOL 28 | 17.21±2.75 | 23.95±4.06 | <0.001^***^ |
| UNBCR DOL 7 | 14.57±1.10 | 16.40±2.19 | <0.001^***^ |
| UNBCR DOL 14 | 12.93±1.05 | 15.36±1.63 | <0.001^***^ |
| UNBCR DOL 21 | 11.29±1.16 | 14.00±1.63 | <0.001^***^ |
| UNBCR DOL 28 | 8.72±1.37 | 12.50±1.55 | <0.001^***^ |

^***^*P* <0.001. Abbreviations: BPD, bronchopulmonary dysplasia; DOL, days of life; UNBCR, urinary NT-pro-BNP/creatinine ratio; UDGCR: urinary 8-OHdG/creatinine ratio.

**Supplementary Table 2.** Correlations between UDGCR and UNBCR and the mechanical ventilation duration and oxygen exposure time

| Variable | Mechanical Ventilation Duration | Oxygen exposure time |
| --- | --- | --- |
| UDGCR DOL 7 | 0.197^*^ | 0.234^**^ |
| UDGCR DOL 14 | 0.405^**^ | 0.429^**^ |
| UDGCR DOL 21 | 0.359^**^ | 0.453^**^ |
| UDGCR DOL 28 | 0.336^**^ | 0.444^**^ |
| UNBCR DOL 7 | 0.175^*^ | 0.254^**^ |
| UNBCR DOL 14 | 0.308^**^ | 0.429^**^ |
| UNBCR DOL 21 | 0.291^**^ | 0.423^**^ |
| UNBCR DOL 28 | 0.354^**^ | 0.505^**^ |

^*^*P* <0.05; ^**^*P* <0.01. Abbreviations: DOL, day of life; UNBCR, urinary NT-pro-BNP/creatinine ratio; UDGCR, urinary 8-OHdG/creatinine ratio.
